# Positive biofilms to control surface-associated microbial communities in a broiler chicken production system - a field study

**DOI:** 10.3389/fmicb.2022.981747

**Published:** 2022-08-15

**Authors:** Virgile Guéneau, Ana Rodiles, Bastien Frayssinet, Jean-Christophe Piard, Mathieu Castex, Julia Plateau-Gonthier, Romain Briandet

**Affiliations:** ^1^Université Paris-Saclay, INRAE, AgroParisTech, Micalis Institute, Jouy-en-Josas, France; ^2^Lallemand SAS, Blagnac, France

**Keywords:** positive biofilms, biosecurity, livestock building surfaces, microbial community structure, bacterial pathogens

## Abstract

In the One Health concept, the use of beneficial bacteria to form positive biofilms that prevent the settlement of undesirable bacteria is a promising solution to limit the use of antimicrobials on farms. However, there is a lack of field studies reporting the onset of these beneficial bacteria after application and the effects on autochthonous surface microbiota. In the study reported here, the inner surfaces of commercial broiler chicken houses were treated or not with a bacterial consortium composed of *Bacillus* spp. and *Pediococcus* spp. strains, able to form covering biofilms in different laboratory models. Preinstalled coupons were sampled over time to capture microbial biofilm dynamics on-farm surfaces. The results showed that the bacterial consortium can establish on the farm surfaces, modulate microbial communities, and limit the implantation of Enterobacteriaceae and Enterococcaceae, two families containing potential pathogens.

## Introduction

In standard intensive broiler farms, chickens live all their life on the same floor mainly covered by straw litter, wood shavings, or sawdust. Animal density is high (up to 21 birds/m^2^), and physicochemical conditions (illumination, temperature, and humidity) are optimized to favor animal growth. These conditions obviously trigger the development of microorganisms on the farm surfaces among which potential undesirable microorganisms (Guéneau et al., 2022b). Cleaning and disinfection (C&D) procedures are applied between each production batch in order to eliminate potential pathogens and avoid cross-contamination through a reduction of the microbial load of livestock building surfaces. These procedures are typically composed of wet cleaning with detergent, disinfection, rinsing, and vacancy period steps before new animals enter (Luyckx et al., 2015a). The disinfectants typically used are quaternary ammonium compounds, aldehydes, and alcohols applied on surfaces by spraying or fogging (Luyckx et al., 2015b). It has been shown that these procedures are not totally effective in eradicating surface-associated communities (Luyckx et al., 2015a). The presence of microbial communities spatially organized on surfaces has been invoked to explain the incomplete action of C&D procedures (Flemming and Wuertz, 2019). Those surface-associated communities often named biofilms are three-dimensional microbial structures adhering to the surface and enclosed in self-produced extracellular polymeric substances (EPSs; Flemming, 2011). The EPS composition can vary between biofilms but is typically composed of a complex mixture of water and biopolymers including polysaccharides, eDNA, proteins, and amyloid fibers. Thanks to their spatial structure, the protective effect of the EPS matrix, and cellular phenotypic heterogeneity, biofilms can adapt exceptionally to environmental fluctuation and are often strongly tolerant to the action of antimicrobials (Bridier et al., 2011; Flemming et al., 2016). In their biofilm form, pathogens such as *Campylobacter jejuni, Enterococcus* spp., *Escherichia coli*, or *Salmonella* spp. have been shown to survive C&D procedure, leading to cross-contaminations between batches of animals (Peyrat et al., 2008; Marin et al., 2009, 2011). As an example, the contamination of chickens with the main zoonotic bacterial pathogen *Campylobacter* spp. in Europe can be explained by its ability to form biofilms on surfaces (Trachoo et al., 2002; Newell and Fearnley, 2003; EFSA and ECDC, 2021). Similarly, the major pathogens responsible for zoonosis in Europe have been described as biofilm formers in livestock building (Guéneau et al., 2022b). These observations support the need to study deeper biofilms in animal production systems for their control.

The poultry sector is one of the fastest growing and most flexible livestock sectors representing half of the additional meat expected to be produced within the next 10 years (OECDE and FAO, 2021). The current societal context leads producers to increase the sustainability and the biosecurity of their farms while reducing the use of antimicrobials such as antibiotics and surface disinfectants. In addition to polluting the environment, nonspecific and abusive use of these antimicrobials triggers the emergence of resistance that can be carried by pathogenic agents (McEwen and Collignon, 2018). Thereby, the World Health Organization defines antimicrobial resistance as a world public health threat that has to be managed urgently. In order to prevent zoonoses and increase food safety, innovative biosecurity tools are implemented. Biosecurity procedures are a set of measures designed to protect against the entry and spread of pathogens. The use of bacteria able to form positive biofilms and guide the microbial ecology of surfaces after C&D procedures is a new and promising biosafety tool. The concept is based on the rapid onset of beneficial bacteria that will occupy ecological niches on surfaces. These beneficial bacteria are being selected for their biofilm-forming abilities and other features linked to spatial and nutritional competition (Alvarez-Ordóñez et al., 2019; Guéneau et al., 2022b). Surface bioprotection is already used by breeders, and some products composed of cocktails of beneficial bacteria are already on the market. However, to our knowledge, no scientific study reports their stepwise implantation on livestock surfaces and their effects on the autochthonous microbiota.

In this study, the modulation of surface-associated microbial communities by the addition of a product composed of a consortium of selected bacteria was studied. First, biofilm phenotypes of the strains that composed the product were studied in different laboratory models. Then, using a field methodology previously described based on preinstalled coupons, surface microbiota was captured over time in treated and untreated buildings (Guéneau et al., 2022c). An integrated global analysis was used to analyze (i) the *in-situ* spatial organization of the surface-attached communities by confocal laser scanning microscopy (CLSM), (ii) bacteria counting in specific agar media, and (iii) the microbial diversity by high-throughput sequencing of the 16S rRNA gene. The exclusion effect of the product on Enterobacteriaceae and Enterococcaceae was studied using relative abundance ratio (Morton et al., 2019). Indeed, these two families contain chicken pathogens of interest such as *Salmonella* spp., *Escherichia coli, Enterococcus faecalis*, and *Enterococcus cecorum* (Foley et al., 2013; Manges, 2016; Souillard et al., 2022).

## Methods

### Macro-colonies, pellicles, and swarming phenotypes

All axenic experiments began with 5 ml of trypticase soy broth (TSB; Biomerieux, France) at 30°C overnight cultures without agitation made from an −80°C glycerol stock. The bacterial consortium LALFILM PRO® (Lallemand SAS, Blagnac, France) was diluted in TSB at 0.4 g/30 ml and was used after agitating for 2 h at 180 rpm at 37°C.

For macro-colonies phenotypes, six-well plates were used with 4 ml of TSA 1.5% agar supplemented with Congo red 40 μg/ml (Sigma-Aldrich, France) to observe amyloid fiber production and 20 μg/ml Coomassie Brilliant Blue to contrast protein production (Sigma-Aldrich, France; Neumann et al., 1994; Jones and Wozniak, 2017). Around 3 μl of culture was deposited in the center of each well, left to dry for 10 min, and then incubated at 30°C for 6 days. For pellicle formation, 4 ml of TSB was inoculated in the same conditions and incubated for 2 days at 30°C.

To perform the swarming experiments, TSB plates supplemented with agar to obtain 0.8% final were prepared (TSB Agar, Biomerieux, France) and allowed to dry in a hood for half an hour. Around 10 μl of culture was deposited in the center of the plate and left to dry for 10 min before 1 day of incubation at 30°C. For each model, a representative picture from three biological replicates was selected.

### Field experiments in livestock buildings

Two independent experiments were conducted in France (batch 1 and batch 2), each with a building treated with a positive biofilm and an untreated control building. Each commercial broiler chicken house building was 800 m^2^ (one control building and one treated per batch). The buildings had identical and unconnected artificial cross-ventilation systems and contained ~18,000 chickens each. Batch 1 was the experiment performed in summer 2020, and batch 2 was the one performed in winter 2020. Buildings of the two batches have never been treated with positive bacteria before.

Similar C&D procedures were performed in the buildings before experiments. They consist of cleaning the building with water the day after the animals left and applying the HD4N detergent (Anti-Germ Deutschland GmbH, Memmingen, Germany) at 1 ml/L, followed by rinsing with water under pressure. Calcium oxide was then applied the next day at 500 g/m^2^ followed by treatment with VIROCID disinfectant (CID lines, Ypres, Belgium) at 1 ml/L. Around 4 kg/m^2^ of chopped straw was set up without new addition during the batch, and then a fumigation protocol with FUMAGRI OPP (LCB Food Safety, Boz, France) was done.

A recently developed protocol was used to study farm surface-associated microbiota (Guéneau et al., 2022c). Briefly, polyvinyl chloride (PVC) coupons were cut from a flat bar (LEROY MERLIN, France) to obtain dimensions of 2.5 cm × 6 cm × 3 mm. Coupons were sterilized in an autoclave (HMC EUROPE, Germany), and dried in a dry oven (FD 115 model, Binder, Germany) for 15 min at 120°C. Then, they were deposited after the C&D procedure 4 days before animal entry below the central water lines of the buildings. The 1st day of the experiments corresponded to the day when coupons were deposited. LALFILM PRO® (Lallemand SAS, France) (total count minimum is 2^10^ CFU/g) was resuspended in tap water and applied following the commercial instructions at a rate of 0.2 g/m^2^. The application was performed with a low pressure (<4 bars) atomizer on all farm internal surfaces including litter, walls, ceiling, drinkers, and feeders without chickens inside the building. The same procedure was applied only with tap water in the control building. The coupons were collected over time, this is five coupons per condition and per sampling day with sterile gloves and analyzed.

### Confocal laser scanning microscopy

Biofilm structures on coupons were observed using a Leica HCS-SP8 CLSM at the INRAE MIMA2 microscopic platform (https://doi.org/10.15454/1.5572348210007727E12).

Environmental biofilms from coupons were labeled with 50 μl of a 54 μM calcein acetoxymethyl (CAM) solution (metabolic fluorescent dye reporting esterase activity). The dye was poured on the coupons and incubated in dark for 30 min at 37°C (Invitrogen, Carlsbad, CA, USA). The non-ionic molecules can enter passively into cells and be cleaved by intracellular esterase releasing a fluorescent non-permeant ionic residue. Biofilms on the coupons were counter-labeled in red with 50 μl of a 3 μl/ml of SYTO 61 (Invitrogen, Carlsbad, CA, USA), a cell-permeant red dye that labels nucleic acid.

For submerged *in vitro* biofilms, 1/100^e^ dilution in TSB was performed from the overnight cultures of strains alone or a suspension of the product concentration in TSB of 0.4 g/30 ml placed in a 50-ml Falcon after agitating for 2 h at 37°C. In total, 200 μl of the solutions were poured into the wells of polystyrene 96-well microtiter plates with a μclear® base (Greiner Bio-one, France) for 1.5 h at 30°C for an adhesion step. Supernatants were removed, 200 μl of fresh media were added, and the plate was put at 30°C for 2 or 24 h. A solution of 3 μl/ml of SYTO 9, a cell-permeant green dye that labels nucleic acid (Invitrogen, Carlsbad, CA, USA) was prepared, and 50 μl of this solution was added to each well.

A 600-Hz frequency was used to acquire images with the CLSM. SYTO 61 was excited with the HeNe laser at 633 nm, and the emitted fluorescence was collected with a hybrid detector in the range of 650–700 nm. SYTO 9 and CAM were excited with an argon laser set at 488 nm, and the emitted fluorescence was collected with a hybrid detector in the range of 500–550 nm. For all acquisitions from this work, a series of four images for each coupon of 512 × 512 pixels was acquired using a 63x water objective lens (numerical aperture = 1.2) by taking one image per μm in Z to capture the full height of the biofilm.

The 2D projections of biofilms and the extracted biofilm biovolume (μm^3^/μm^2^) were obtained using IMARIS 9.3.1 software (Bitplane, AG - Zurich, Switzerland).

### Enumeration of culturable microorganisms from coupons

Coupons were placed in individual tubes containing 30 ml of a saline solution (NaCl 9 g/L). With a pipette cone, the biofilm was mechanically disrupted by successive round trips. Twenty passages vertically and horizontally were made on both sides of the coupon. After homogenization by vortexing 5 s and pipetting, successive dilutions in saline solution were carried out in duplicate using 1 ml of the resuspended biofilm solution. Counts into agar were made from 1 ml of the desired dilution. TSA (Biomerieux, France) was used as nonselective media under aerobic conditions for 24 h at 30°C. In order to estimate bacilli spores in environmental biofilms, 1 ml of detached biofilm suspension was placed in a glass tube that was immersed in a water bath for 10 min at 80°C, in duplicate for each coupon, before enumeration on TSA (“TSA 80°C” condition). PSA+A medium (MRS, supplemented with cysteine hydrochloride 0.05% (wt/vol) + 100 μg/L novobiocin + 10 mg/L vancomycin + 50,000 U/L nystatin + 1 mg/L ampicillin) was used to enumerate *Pediococcus* spp. (Simpson et al., 2006). The remaining 26 ml of the detached biofilm suspension was centrifuged for 10 min at 6,000 × *g*, the supernatant was gently removed, and the pellets were placed at −20°C for DNA extraction.

### High-throughput sequencing of the 16S rRNA gene and diversity analysis

#### DNA extraction, PCR, and sequencing

DNA from 80 bacterial pellets was extracted using DNeasy PowerLyzer PowerSoil Kit following the manufacturer's instructions (Qiagen, Germany). For PCR strategy, the same methodology was used as reported in Guéneau et al. (2022c). In short, this is PCR of V3-V4 regions of 16S rRNA marker genes using Phusion High-Fidelity PCR Kit (New England Biolabs, UK) amplified with universal primers F343 (5-CTTTCCCTACACGACGCTCTTCCGATCTTACGGRAGGCAGCAG-3) and R784 (5-GGAGTTCAGACGTGTGCTCTTCCGATCTTACCAGGGTATCTAATCCT-3) at 66°C of annealing temperature on a thermocycler (GeneAmp PCR System 9700, Applied Biosystems, USA; Verschuren et al., 2018). Around 1% of agarose gel electrophoresis was used to ensure the expected size of the amplicons in PCR products, including negative controls. The NanoDrop Spectrophotometer ND-1000 (Thermo Fisher, Waltham, MA, USA) was used to quantify the DNA. DNA amplicons were sequenced using Illumina MiSeq technology in the GeT-PlaGe INRAE platform (Toulouse, France).

#### Diversity and taxonomical analysis using bioinformatics

Paired-end fastq files were truncated and denoized with DADA2 (Callahan et al., 2016) under default parameters excluding primers length. *De novo* multiple sequence alignment was performed by fast Fourier transform (MAFFT; Katoh and Standley, 2013), and FastTree was used to construct the phylogeny (Price et al., 2010). Rarefaction curves as goods coverage and observed AVSs were studied to ensure a full sampling of the community was taken. Data were rarefied at sequence depth higher than 10,000 sequences per sample to study diversity. Alpha-diversity was studied with the Shannon index and beta-diversity using weighted UniFrac distances. Relative abundance and natural log ratios were used to analyze relevant taxonomical changes (Morton et al., 2019). QIIME2 (v2020.2) was used to obtain all bioinformatics results (Bolyen et al., 2019).

#### Statistical analysis

Results are represented by the average and standard deviation (SD) or confidence interval (CI) of five coupons per Day. “Coupon” was considered the experimental unit and treatment the fixed factor. A two-way ANOVA using the uncorrected Fisher's least was used for the count and biovolume analysis using PRISM software (GraphPad, USA, California) with treatment and time as fixed factors. Linear discriminant analysis (LDA) effect size (LEfSe; Segata et al., 2011) was used to identify significant differences in taxonomical relative abundances. Diversity profiles were analyzed with the Kruskal–Wallis, Spearman's correlation, and ANOSIM in QIIME2, and log ratios with the Mann–Whitney *U*-test with seaborn (v0.5.0) in Python (v3.7.6). Data were considered significant when a *p*-value was smaller than 0.05.

## Results

### Beneficial bacteria biofilm phenotypes

**Figure 1:**
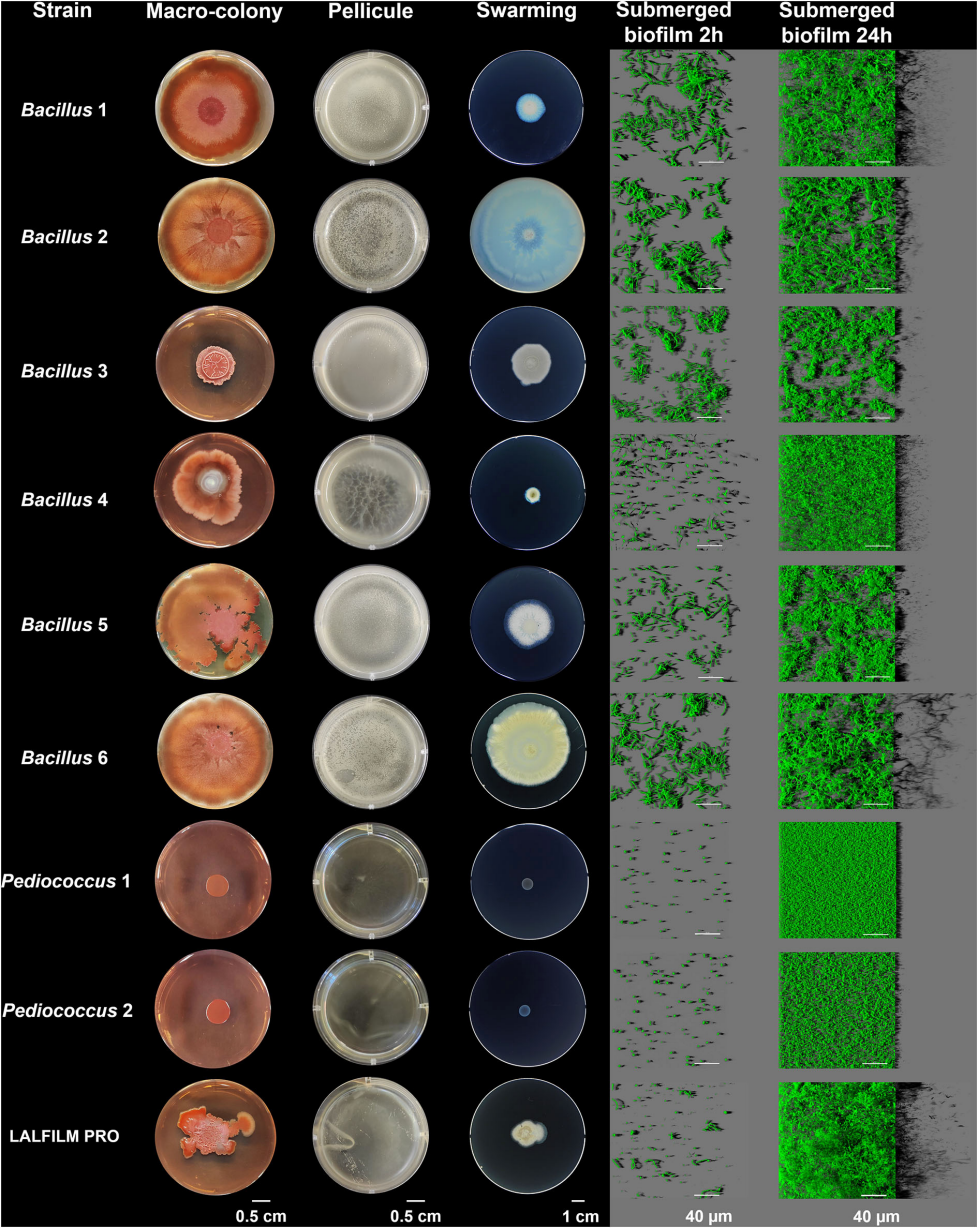
Biofilm phenotypes of the strains composing the bacterial consortium. Macro-colonies were used as a biofilm model at the solid–air interface and pellicles for the liquid–air interface. The swarming model allowed us to identify whether bacteria can colonize semisolid surfaces. Biofilm development of the submerged biofilm at 2 and 24 h using SYTO 9 to label the bacterial cells was observed using CLSM.

The positive biofilm applied to building surfaces was composed of six strains of *Bacillus* spp. and two strains of *Pediococcus* spp. Four laboratory biofilm models were used to study the phenotypes of the strains composing the positive biofilm bacterial consortium (Figure 1). In the macro-colony model, which is an agar–air interface biofilm model, *Bacillus* 3 was able to form a structured colony with wrinkles, whereas the other *Bacillus* spp. have a flat structure covering most of the Petri dish. The bacterial consortium forms wrinkles and a beginning of spread is observed on the agar plate. In contrast to *Pediococcus* spp., *Bacillus* spp. strains and the bacterial consortium were able to form pellicles at the liquid–air interface.

In a swarming model on semisolid-air, *Bacillus* 2 was able to colonize the totality of the Petri dish after 24 h. *Bacillus* 4 and *Pediococcus* spp. were not able to swarm.

A submerged biofilm model was studied using CLSM. At 2 h, all strains started to cover the surface. The bacterial consortium showed a diversity of cell morphologies with cocci and bacilli. At 24 h, *Bacillus* 6 formed a spectacular thick biofilm as visualized by the virtual lateral shadow projection. The other strains covered the majority of the surface of wells with a thin layer of bacteria. The LALFILM PRO® showed a structured and dense biofilm structure.

### A stepwise installation of biofilms on farm surfaces

#### Microscopic quantification of the biofilm formation on coupons

**Figure 2:**
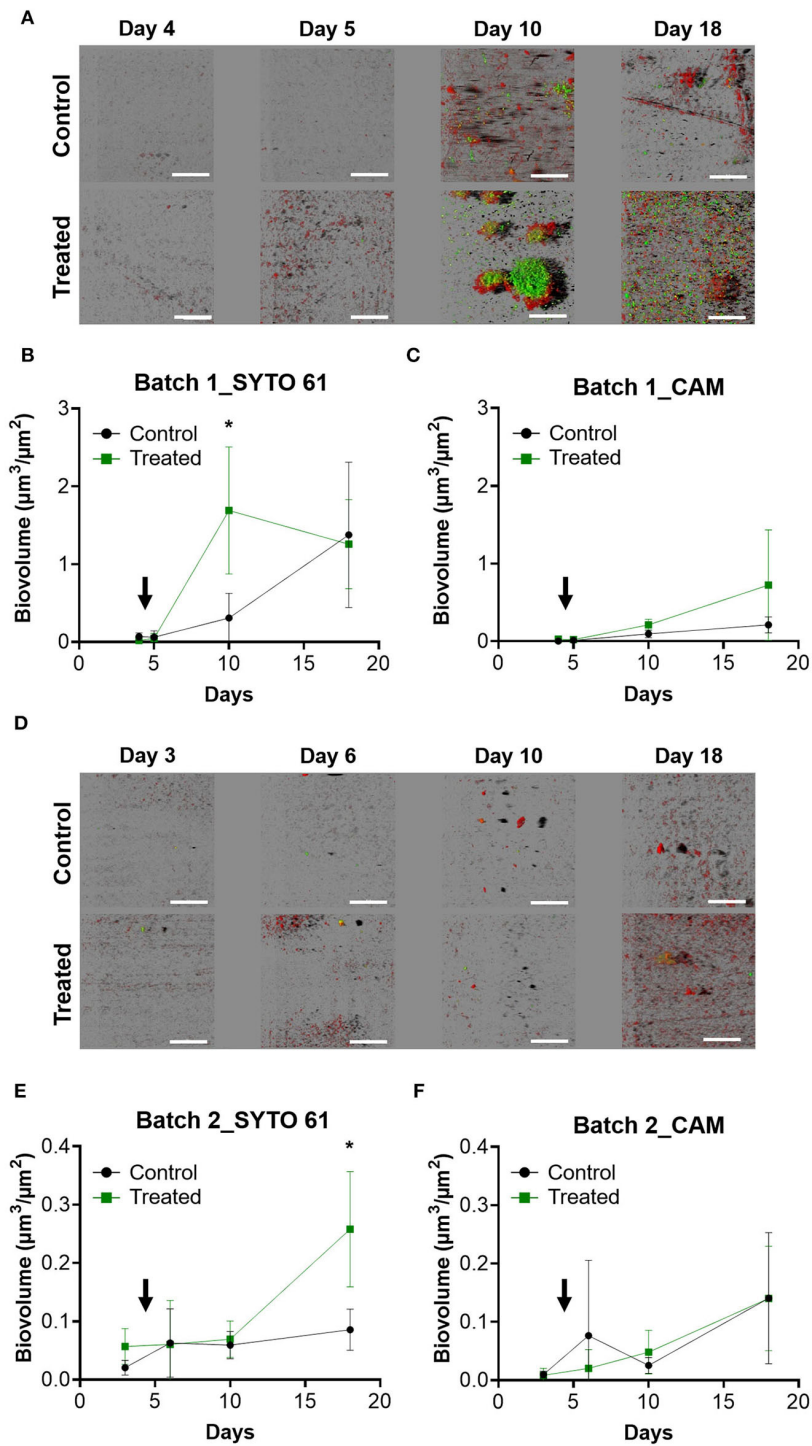
Visualization and quantification of surface-attached microbial communities of farms depending on the treatment and during the time. After sampling, coupons were labeled with SYTO 61 to detect the entire population in red and with CAM to contrast metabolically active populations and then observed with CLSM. IMARIS software was used to visualize images in blend mode **(A,D)** and to quantify SYTO 61 biovolume for batch 1 **(B)** and batch 2 **(E)**. Similarly, quantification of CAM was performed for batch 1 **(C)** and batch 2 **(F)**. The black arrow indicates the day when the animals enter the farm. Error bar shows standard deviation and asterisks represent significant differences between conditions on the same day (*p* < 0.05).

Visualization of biofilms that developed on the surface of coupons was performed using CLSM. In batch 1, the day before the entrance of animals, a few microorganisms were detected, but the images show structured biofilms from day 10 with green clusters corresponding to metabolically active bacteria (CAM), especially in the treated condition (Figure 2A). Biovolume showed significantly more SYTO 61 signals corresponding to the entire microbial population on day 10 in the treated condition (Figure 2B). CAM biovolume signals showed no significant differences between the control and the treated condition (Figure 2C).

The surfaces of the second batch were less colonized by biofilms compared to the first batch, and no structured biofilm was observed for all time points (Figure 2D). On day 18, a higher SYTO 61 biovolume was detected in the treated condition (*p* < 0.05; Figure 2E).

As in batch 1, no significant differences with CAM biovolume were observed between the control and the treated conditions (Figure 2F). The range of biovolume values for SYTO 61 and CAM was 10 times smaller in batch 2 compared to batch 1.

#### Enumeration of cultivable bacteria

**Figure 3:**
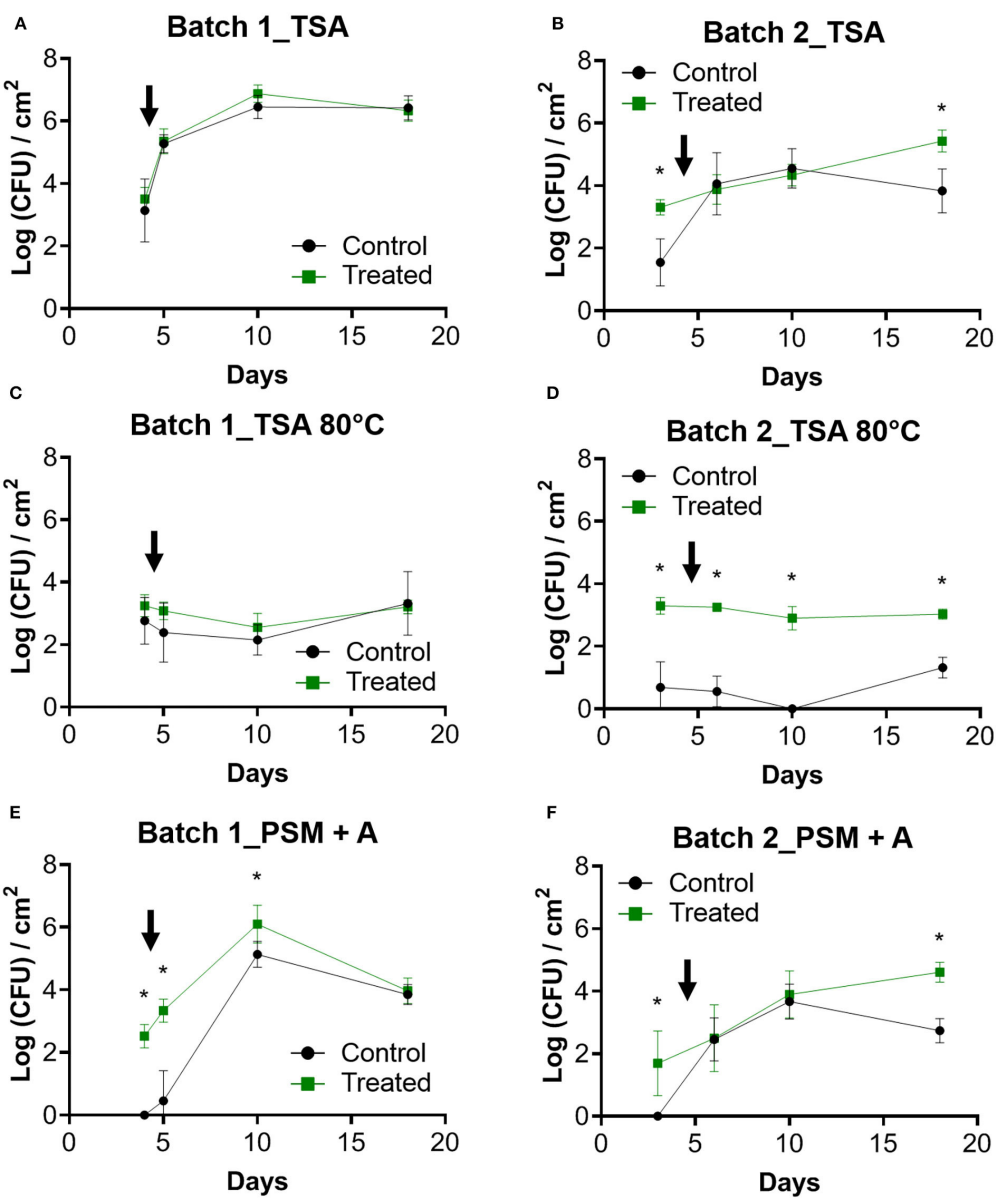
Enumeration of bacteria from coupons. Counts over time were performed on the biofilms previously removed from the coupons for batch 1 **(A,C,E)** and batch 2 **(B,D,F)**. TSA was used as a nonselective medium. Enumerations of these samples on TSA after treating them for 10 min at 80°C were performed to select spores forming bacteria. PSM + A medium was used to count *Pediococcus* genus. The black arrow indicates the day when the animals enter the farm. Error bar shows standard deviation and asterisks represent significant differences between conditions on the same day (*p* <0.05).

In the first batch, TSA counts of the control and the treated condition did not differ significantly across the experimental period (Figure 3A). An increase in TSA counts was measured after the entry of the animals and subsequently stabilize on day 10, reaching more than 6 logs (CFU)/cm^2^. Initial values on TSA differed between conditions in batch 2 with significantly higher counts in the treated condition (*p* < 0.05; Figure 3B). A linear increase of CFU was observed in the treated condition, and a decrease appears between days 10 and 18 compared to the control (*p* <0.05).

No significant differences between the two conditions of batch 1 were determined for TSA 80°C counts (Figure 3C). No increase in spore counts was observed after animal entry. In batch 2, more spores than the control were counted in the treated condition (*p* <0.05; Figure 3D). A significantly lower number of spores was determined in the control of batch 1 compared to the control of batch 2 (*p* <0.05). Interestingly for the two batches and in both conditions, the spore count values were very stable over time.

PSM + A counts were significantly higher in treated conditions for all samples except for day 18 in batch 1 (Figure 3E). For batch 2, the counts increased progressively in both conditions, whereas a decrease was observed on day 18 in the control condition (Figure 3F). With PSM + A medium, nothing was counted at the initial time point for the control condition of the two batches, whereas around 2 logs were observed in the treated conditions (*p* <0.05; Figures 3E,F).

### Modulation of livestock biofilm communities composition by the addition of beneficial bacteria

In total, 80 samples were successfully sequenced producing a total of 4,067,961 sequences of 411 ± 26 bp. After denoizing, a total of 1,997,152 corresponding to 2,187 ASVs were kept. Rarefaction curves showed a *plateau* and goods coverage close to 1 from about 5,000 sequences (Supplementary Figure 1). Two samples were discarded for the diversity analysis as <10,000 reads were recovered (3,760 and 5,316 sequences). Mitochondria and chloroplast were also removed (8 ASVs in total corresponding to 1,201 sequences) before the downstream taxonomical analysis.

Weighted UniFrac distances were studied by using ANOSIM (Table 1). For batch 1, distances between treatments were significantly different on days 4, 10, and 18 (*p* <0.05). For batch 2, distances between treatments were significantly different on days 3, 10, and 18 (*p* <0.05), with a trend for day 6 (*p* = 0.068).

**Table 1:** ANOSIM of batch 1 and batch 2 per sampling day.

		**Batch 1**		**Batch 2**	
		***R* statistic**	***p*-value**	***R* statistic**	***p-*value**
Global	0.603	0.001*	0.700	0.001*
Pairwise (control vs. treated)	Day 3			0.969	0.011*
	Day 4	0.506	0.025*		
	Day 5	0.08	0.207		
	Day 6			0.280	0.068
	Day 10	0.404	0.019*	0.564	0.007*
	Day 18	0.212	0.032*	0.644	0.008*

*Significant differences between control and treated groups (p <0.05).

**Figure 4:**
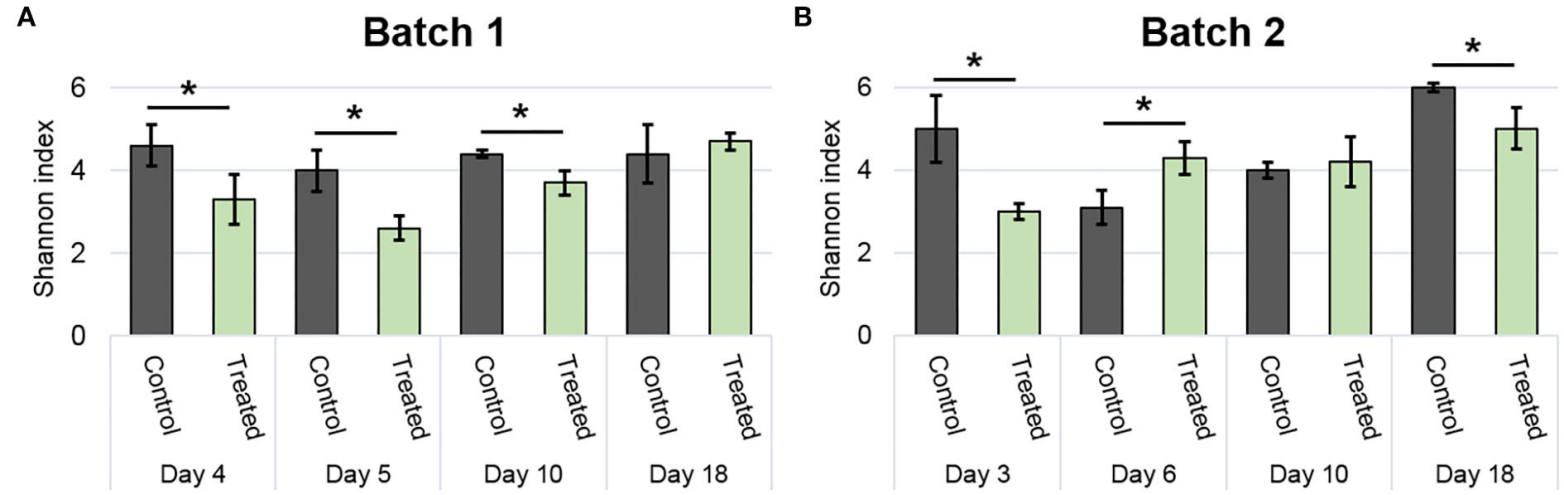
Modulation of bacterial community diversity by the addition of positive bacteria. The Shannon index of batch 1 **(A)** and batch 2 **(B)** during the experimental period. Error bar shows standard deviation and asterisks represent significant differences between conditions on the same day (*p* <0.05).

The Shannon index was used to compare alpha-diversity between conditions (Figure 4). In general, the Shannon index was stable over time, specifically in batch 1 (Figure 4A). Lower values than control were observed in the treated condition for days 4, 5, and 10 in batch 1, and on days 3 and 18 in batch 2 (*p* <0.05; Figure 4B). However, an increased Shannon diversity was noticed in treated vs. control on day 6 of batch 2 (*p* <0.05; Figure 4B).

At phyla level 4, phylum described coupons composition as: Firmicutes (57.6%), Proteobacteria (38.6%), Actinobacteria (3.1%), and Bacteroidetes (0.6%; Supplementary Figure 2). The evolution of the phyla over time showed a predominance of Firmicutes (66.2%) over Proteobacteria (31.1%) and Actinobacteria (2.7%) before entry of the animals (day 3 or 4), then similar proportions of Firmicutes (51.7%) over Proteobacteria (47.4%) from day 5/6 to day 10, and finally a predominance of Firmicutes (64.5%) over Proteobacteria (25.7%), Actinobacteria (7.8%), and Bacteroidetes (2.1%) at day 18. When comparing conditions per batch, significant differences were found mainly in batch 2 on day 3 with a higher abundance of Firmicutes in the treated group and a higher relative abundance of Proteobacteria and Actinobacteria in the control (*p* <0.05).

**Figure 5:**
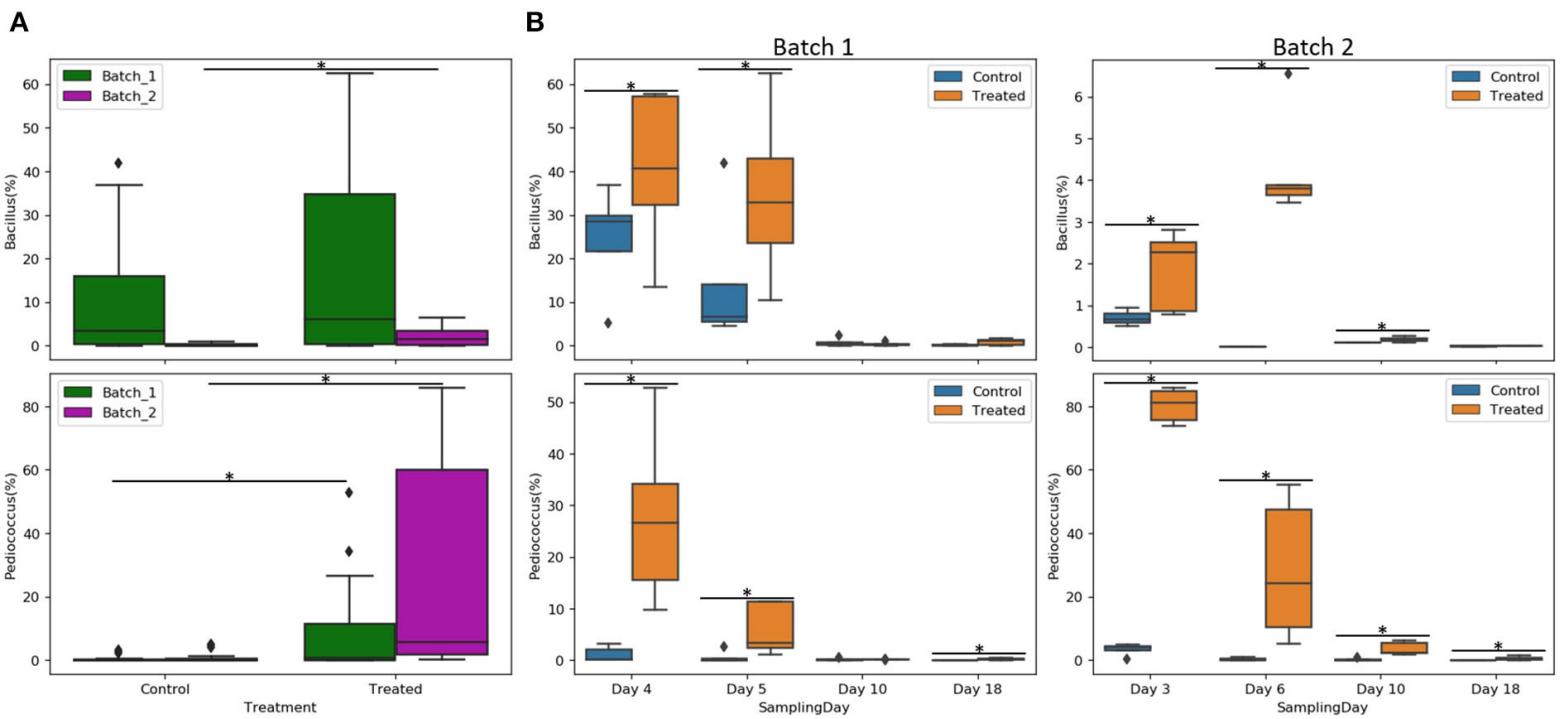
Relative abundance of *Bacillus* and *Pediococcus* per batch **(A)** and per sampling day **(B)** of different conditions (control or treated). Asterisks represent significant differences between conditions (*p* <0.05).

Individual sample profile at the family level reveals a relatively small variability across all five coupons per day and condition (Supplementary Figure 3). The entry of the animals led to increases in the relative abundance of Enterobacteriaceae and Enterococcaceae families and decreases in Lactobacillaceae and Bacillaceae families. The relative abundance of *Bacillus* spp. and *Pediococcus* spp. and relevant ratios were analyzed. For both genera, abundance profiles across the experimental period differed between batches and treatments (Figure 5A). *Pediococcus* spp. relative abundance was significantly higher in the treated group for all days (*p* <0.05), except on day 10 of batch 1, whereas *Bacillus* spp. relative abundance in the treated condition was higher compared to the control on days 4 and 5 for batch 1 and days 3, 6, and 10 for batch 2 (*p* <0.05; Figure 5B). In the same way, the Enterobacteriaceae family was reduced on day 10 for batch 1 and on day 18 for batch 2 in the treated condition compared to the control (*p* <0.05); Enterococcaceae family was also reduced on days 3, 6, and 18 for batch 2 in treated condition compared to control (*p* <0.05; Supplementary Figure 4).

**Figure 6:**
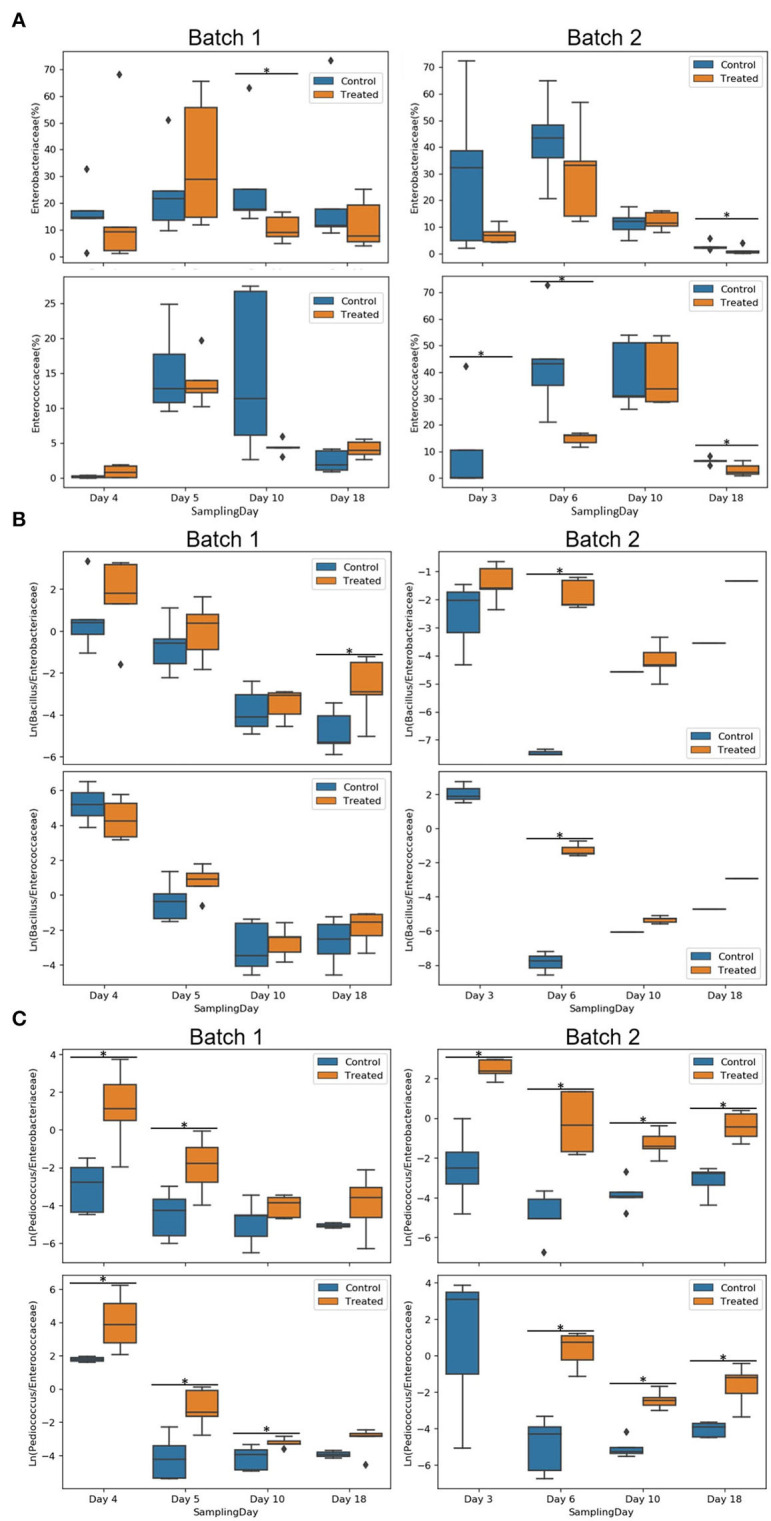
Comparison of Enterobacteriaceae and Enterococcaceae families against *Pediococcus* spp. and *Bacillus* spp. using natural log ratio. Relative abundance of Enterobacteriaceae and Enterococcaceae of batches 1 and 2 per condition (control and treated) and sampling day **(A)**. Natural log ratios of *Bacillus* **(B)** or *Pediococcus* **(C)** vs. Enterobacteriaceae or Enterococcaceae on the coupon biofilms of batch 1 and batch 2 per condition (control or treated) and sampling day. Sample representation per ratio was 76 biofilm coupons for *Pediococcus*/Enterobacteriaceae, 64 for *Bacillus*/Enterobacteriaceae, 66 for *Pediococcus*/Enterococcaceae, and 55 for *Bacillus*/Enterococcaceae coupons. Note that no Enterococcaceae was present in the treated condition on day 3 (infinite ratio), and other samples contained zeros on one side of their log ratio. Asterisks represent significant differences between conditions (*p* <0.05).

Enterobacteriaceae and Enterococcaceae families were compared against *Pediococcus* spp. and *Bacillus* spp. using natural log ratio (Figure 6). There was no Enterococcaceae detected on day 3 in the treated group of batch 2 at the earliest time point, making the ratio infinite on that day (not shown in plots; Figure 6A). Natural log ratio (*Bacillus*/Enterobacteriaceae) was enhanced by the treated condition, which was globally significant for batch 2 (Supplementary Figure 4) and specifically significant on days 18 and 6, respectively, for batches 1 and 2 (*p* <0.05; Figure 6B). Similarly, treated conditions showed increases in natural log ratio (*Pediococcus*/Enterobacteriaceae) for all days with statistical significance for the first days (3–6 days) for both batches. At later time points, the same effect was significantly detected on days 10 and 18 of batch 2 (Figure 6C). Natural log ratio (*Pediococcus*/Enterococcaceae) in treated conditions was always higher vs. control, but significantly higher from day 3/4 to day 10 in batch 1 and until day 18 in batch 2 (*p* <0.05; Figure 6C).

## Discussion

The objective of this work was to analyze the modulating effect of a mixture of *Bacillus* spp. and *Pediococcus* spp. strains on the natural surface-attached microbial communities associated with poultry houses. Before testing in field conditions, several biofilm models were used in the laboratory to characterize the traits of the bacteria that have been applied on the farm. All the strains were able to form biofilms, and the combination of all of them constitutes the LALFILM PRO® product, which can form covering and structured biofilm *in vitro*. The set of complementary techniques used to analyze livestock building surfaces demonstrated that the mixture of beneficial bacteria can also be established on farm surfaces. It also revealed contrasted situations and heterogeneities between farm buildings (batches). Indeed, even if the observed environmental biofilms of the farm were sparse, quantification of the SYTO 61 biovolume allowed to show that more biomass was detected in the treated condition in both batches. Specific counts showed more *Pediococcus* spp. in the treated conditions, especially the first few days of sampling before the animals entered, with no *Pediococcus* spp. detected in the controls. Relative abundances of *Pediococcus* spp. were significantly higher in the treated condition for both batches. The large amount of *Bacillus* spp. detected in batch 1 control makes it difficult to differentiate the beneficial bacilli from the mixture that was applied in the treated building. In batch 2, significantly more total aerobic bacteria were counted in the treated condition, and *Bacillus* spp. relative abundance correlated with the spore counts on TSA 80°C for all time points, except for day 18.

In addition to being implanted on surfaces, the product can modulate the alpha diversity of the surface communities. In batch 1, a decrease of the Shannon index is observed for the treated group on days 4, 5, and 10 before returning to the same value as the control. Since the Shannon index is calculated based on species richness and evenness, adding a large number of bacteria of identical species lowers the value. These values are in line with the establishment of the products on the surfaces at least for the 1st days. The same observations were made for batch 2 on days 3 and 18, but a lower Shannon index value is calculated in the control on day 6, which could be explained by a higher relative proportion of Enterobacteriaceae (43%) and Enterococcaceae families (43%; e.g., *E. casseliflavus* and *E. cecorum*) due to animal entrance. As revealed by the weighted UniFrac distances of beta diversity, the distribution or composition of species was also modulated by the treatment in both batches and at all sampling points except the next days of animal entry (days 5–6) where a similar composition was found between coupon treatments.

The increase of *Bacillus* spp. and *Pediococcus* spp. abundance was correlated with a decrease in the Shannon diversity. The treatment increases *Bacillus* spp. and *Pediococcus* spp. quantities (i.e., abundance and counts) and the relevant ratios, probing a concomitant decrease of Enterobacteriaceae and Enterococcaceae families. Despite these promising results, further analysis would be required for a better resolution. Other techniques, such as qPCR, will be used in the future to track targeted pathogenic strains (Postollec et al., 2011).

The different approaches showed colonization of the applied consortium, but it is unclear whether the product is able to grow and be metabolically active on the surfaces and not just persistent “without growth” acting, hence more like a physical barrier as the main mode of action. No significant differences were observed with the quantification of CAM biovolume for both conditions and in both batches. More *Pediococcus* spp. were enumerated during the earlier days in the batches in the treated condition, but the increase in values that follow may be due to the detection of environmental *Pediococcus* spp. detected also in the controls. Moreover, the values of spores count were very stable during the experiment even in the control, which makes it impossible to conclude whether the *Bacillus* spp. of the product, initially used in the form of spores, were able to grow. The relative abundance of the *Bacillus* spp. that decreases and the stable value of spores over time are consistent with the idea that the quantity of *Bacillus* spp. is stable during the time on the farm after the C&D protocols. The litter used in batch 1 was left outside for a longer period, which may have resulted in *Bacillus* spp. colonization. This could explain the higher number of spores detected in the batch 1 control compared to batch 2. Another hypothesis is that the C&D process before batch 1 was less efficient because the counts on TSA were also higher in the control building of batch 1 compared to batch 2.

Our results indicate that the initial situation between the two batches was different in terms of community profiles, microbial density, and spatial organization of the communities. Initial counts before animal entry were not identical across batches for control. The bacterial count and biovolume of the batch 1 control were higher than those of the batch 2 control. The CLSM images showed that at the beginning of the rearing cycle, biofilms were poorly developed and did not completely cover the surfaces of the coupons, especially for batch 2. Ten times less biovolume on batch 2 was quantified compared to batch 1. More total bacteria and spores were counted in batch 1 for all the experiments, with a very stable value of spores during the time for the two batches. The number of spores in the control of batch 1 was correlated with the relative abundance of *Bacillus* spp. These results show the importance of biological replicates for this type of field study. The biofilms of the product did not cover the entire surface of the coupons in this farm setting, although it was the case *in vitro*, in the laboratory. It can be interesting to develop a field model in the lab to study biofilm formation in conditions closer to those encountered on farms (temperature, humidity, poor nutritive medium, and materials). Modeling the interactions and testing the ecological theories within these positive bacterial consortia in simplified communities appear to be a promising step to improve the product composition (De Roy et al., 2014). In addition, the study of bacteria naturally present in farms would allow new selection criteria of strains (phenotypes and genomes), already identified as capable of living in these environments (Guéneau et al., 2022a).

A previous study compared the use of beneficial bacteria guiding surface microbial ecology with conventional C&D protocols in livestock buildings (Luyckx et al., 2016). In our study, the application of positive biofilm is a complement to the C&D protocol and not a simple substitution. Indeed, a surface with a reduced load of microorganisms obtained by C&D will be more favorable to the settlement of a positive biofilm. The observation of a decrease in the abundance of *Bacillus* spp. and *Pediococcus* spp. over time suggests that the product will be particularly effective at the beginning of the breeding process, when the microbial load is low, thus limiting the implantation and early development of harmful microorganisms. Other studies showed that natural positive resident microbiota of smear cheese wooden shelves had an effect on the exclusion of pathogens on surfaces by nutritional competition when the natural microbiota is already established or develops faster than the pathogen (Guillier et al., 2008). In addition, studies on *Agaricus bisporus* biocontrol have shown that the addition of positive biofilm-forming bacteria in compost with a low initial microorganism load was able to limit the early establishment of the main green mold mushroom pathogens (Pandin et al., 2019).

## Conclusion

This field study demonstrated that the positive biofilm-forming bacteria used can establish on livestock building surfaces and to modulate the biofilm community structure and diversity, with special reference to the reduction of the ratio involving Enterobacteriaceae and Enterococcaceae families. Large-scale field experiments will be required to get significant statistics on the effect of positive biofilm on the prevalence of specific targeted pathogens. This study also highlights microbial variability between production batches of the same farm within the same building. These promising results encourage the innovative use of positive biofilms to guide these ecological systems.

## Data availability statement

The datasets presented in this study can be found in online repositories. The names of the repository/repositories and accession number(s) can be found at: https://www.ncbi.nlm.nih.gov, PRJNA855604.

## Author contributions

VG, J-CP, BF, MC, JP-G, and RB: conceptualization and methodology. J-CP, MC, JP-G, and RB: validation and supervision. VG and AR: formal analysis and data curation. VG, JP-G, and RB: investigation. MC and RB: resources, project administration, and funding acquisition. VG: writing the original draft preparation. AR, MC, JP-G, and RB: writing, reviewing, and editing. All authors have read and agreed to the published version of the manuscript.

## Funding

This research was funded by INRAE, Lallemand SAS, and Association Nationale de la Recherche et de la Technologie (contract 2020/0548).

## Conflict of interest

Authors VG, AR, BF, MC, and JP-G were employed by Lallemand SAS marketing the product LALFILM PRO® used as a positive biofilm in this study. The remaining authors declare that the research was conducted in the absence of any commercial or financial relationships that could be construed as a potential conflict of interest.

## Publisher's note

All claims expressed in this article are solely those of the authors and do not necessarily represent those of their affiliated organizations, or those of the publisher, the editors and the reviewers. Any product that may be evaluated in this article, or claim that may be made by its manufacturer, is not guaranteed or endorsed by the publisher.
