# Supplementary material for: Positive biofilms to control surface-associated microbial communities in a broiler chicken production system - a field study
Source: Front Microbiol. 2022 Aug 15;13:981747. doi: 10.3389/fmicb.2022.981747 (PMC9421038; doi:10.3389/fmicb.2022.981747)
Supplement: Supplementary file 1 [file Presentation_1.pdf]

Supplementary Material

1 Supplementary Figures

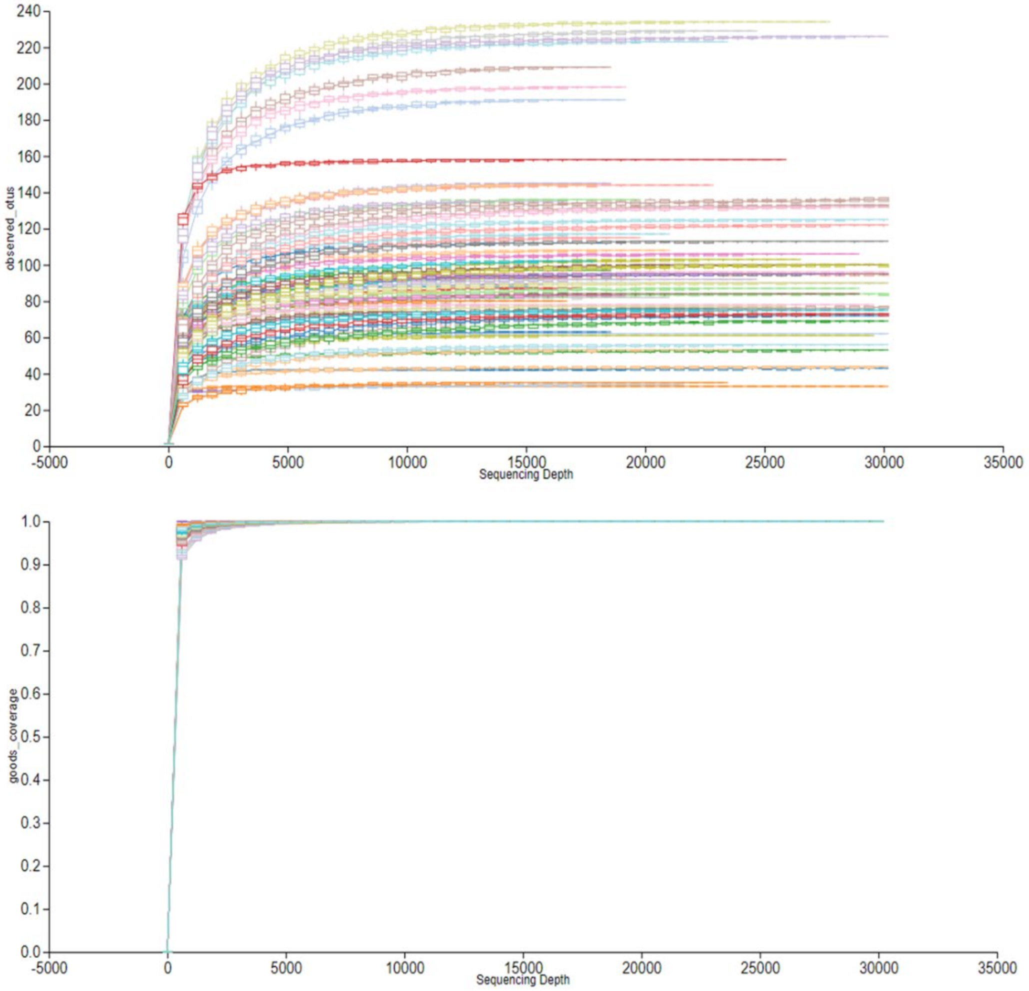

Supplementary Figure 1. Rarefaction plots of observed ASVs and goods coverage for all the coupon biofilm samples.

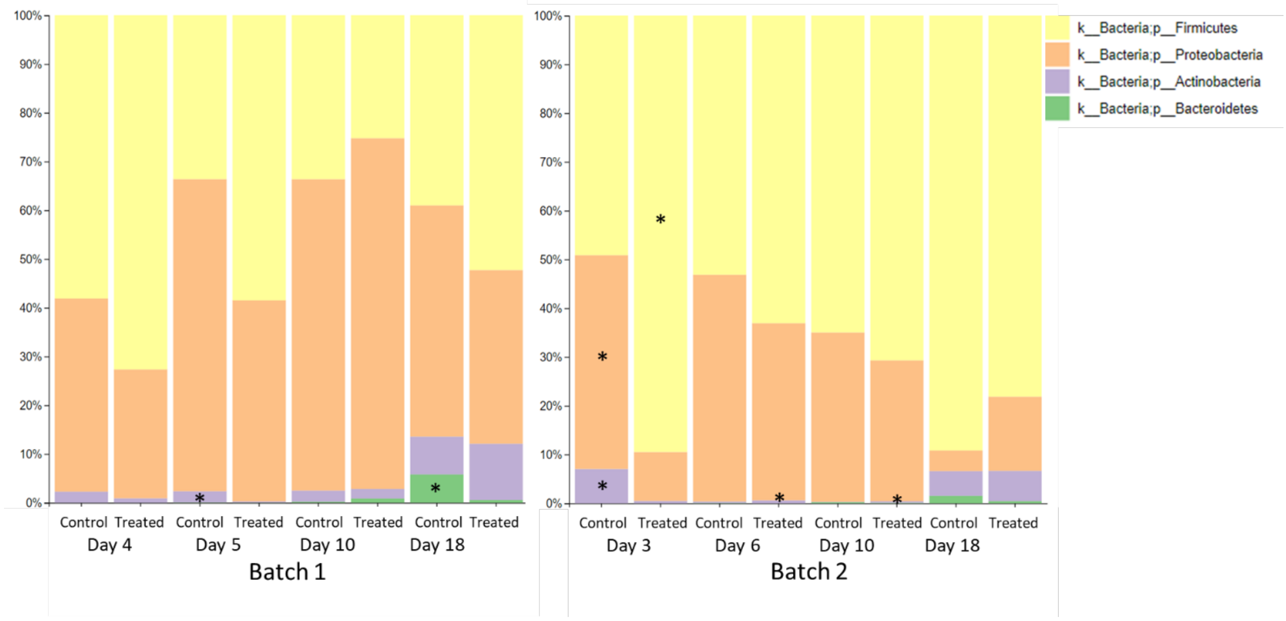

Supplementary Figure 2. Phyla relative abundance (%) for batch 1 and batch 2 per condition (control or treated) and sampling day. Asterisks denotes significant differences between conditions in each sampling day (*P*<0.05).

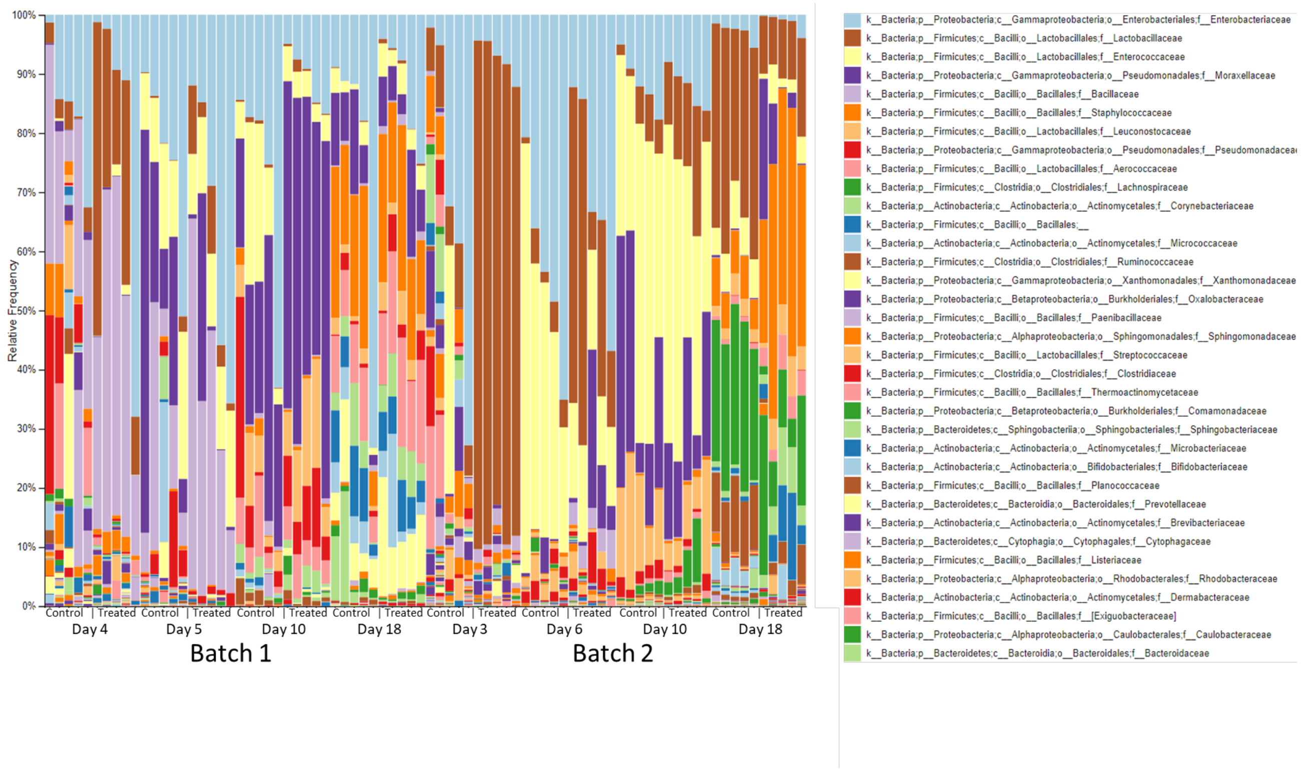

Supplementary Figure 3. Individual coupons relative abundance (%) at family level of batch 1 and 2 per condition (control or treated) and sampling day.

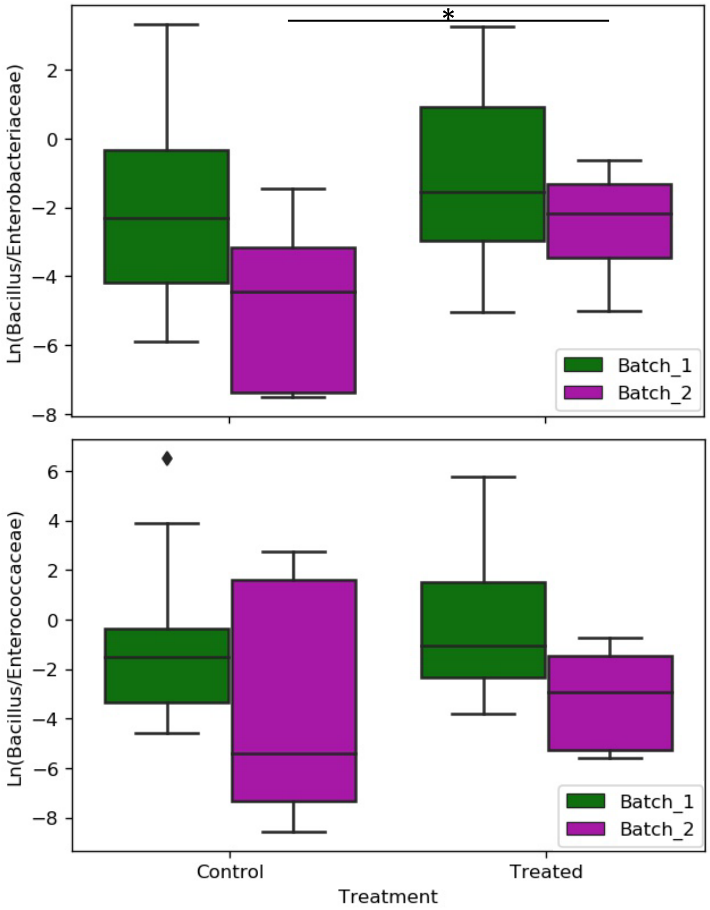

Supplementary Figure 4. Natural log ratios of *Bacillus versus* Enterobacteriaceae or Enterococcaceae on the coupon biofilms of batch 1 and batch 2 per condition (control or treated). Sample representation per ratio was 64 for *Bacillus*/Enterobacteriaceae and 55 for *Bacillus*/Enterococcaceae coupons. Asterisks represent significant differences between conditions (P<0.05).
